# The Transcriptome of the Intraerythrocytic Developmental Cycle of Plasmodium falciparum

**DOI:** 10.1371/journal.pbio.0000005

**Published:** 2003-08-18

**Authors:** Zbynek Bozdech, Manuel Llinás, Brian Lee Pulliam, Edith D Wong, Jingchun Zhu, Joseph L DeRisi

**Affiliations:** **1**Department of Biochemistry and Biophysics, University of California, San FranciscoSan Francisco, CaliforniaUnited States of America; **2**Department of Biological and Medical Informatics, University of California, San FranciscoSan Francisco, CaliforniaUnited States of America

## Abstract

Plasmodium falciparum is the causative agent of the most burdensome form of human malaria, affecting 200–300 million individuals per year worldwide. The recently sequenced genome of P. falciparum revealed over 5,400 genes, of which 60% encode proteins of unknown function. Insights into the biochemical function and regulation of these genes will provide the foundation for future drug and vaccine development efforts toward eradication of this disease. By analyzing the complete asexual intraerythrocytic developmental cycle (IDC) transcriptome of the HB3 strain of P. falciparum, we demonstrate that at least 60% of the genome is transcriptionally active during this stage. Our data demonstrate that this parasite has evolved an extremely specialized mode of transcriptional regulation that produces a continuous cascade of gene expression, beginning with genes corresponding to general cellular processes, such as protein synthesis, and ending with *Plasmodium*-specific functionalities, such as genes involved in erythrocyte invasion. The data reveal that genes contiguous along the chromosomes are rarely coregulated, while transcription from the plastid genome is highly coregulated and likely polycistronic. Comparative genomic hybridization between HB3 and the reference genome strain (3D7) was used to distinguish between genes not expressed during the IDC and genes not detected because of possible sequence variations. Genomic differences between these strains were found almost exclusively in the highly antigenic subtelomeric regions of chromosomes. The simple cascade of gene regulation that directs the asexual development of P. falciparum is unprecedented in eukaryotic biology. The transcriptome of the IDC resembles a “just-in-time” manufacturing process whereby induction of any given gene occurs once per cycle and only at a time when it is required. These data provide to our knowledge the first comprehensive view of the timing of transcription throughout the intraerythrocytic development of P. falciparum and provide a resource for the identification of new chemotherapeutic and vaccine candidates.

## Abstract 2

A tight cascade of gene regulation during the lifecycle of the malaria parasite in human blood cells suggests new functions for many Plasmodium genes

## Introduction

Human malaria is caused by four species of the parasitic protozoan genus *Plasmodium*. Of these four species, Plasmodium falciparum is responsible for the vast majority of the 300–500 million episodes of malaria worldwide and accounts for 0.7–2.7 million annual deaths. In many endemic countries, malaria is responsible for economic stagnation, lowering the annual economic growth in some regions by up to 1.5% (Sachs and Malaney 2002). While isolated efforts to curb malaria with combinations of vector control, education, and drugs have proven successful, a global solution has not been reached. Currently, there are few antimalarial chemotherapeutics available that serve as both prophylaxis and treatment. Compounding this paucity of drugs is a worldwide increase in P. falciparum strains resistant to the mainstays of antimalarial treatment (Ridley 2002). In addition, the search for a malaria vaccine has thus far been unsuccessful. Given the genetic flexibility and the immunogenic complexity of P. falciparum, a comprehensive understanding of *Plasmodium* molecular biology will be essential for the development of new chemotherapeutic and vaccine strategies.

The 22.8 Mb genome of P. falciparum is comprised of 14 linear chromosomes, a circular plastid-like genome, and a linear mitochondrial genome. The malaria genome sequencing consortium estimates that more than 60% of the 5,409 predicted open reading frames (ORFs) lack sequence similarity to genes from any other known organism (Gardner et al. 2002). Although ascribing putative roles for these ORFs in the absence of sequence similarity remains challenging, their unique nature may be key to identifying *Plasmodium*-specific pathways as candidates for antimalarial strategies.

The complete P. falciparum lifecycle encompasses three major developmental stages: the mosquito, liver, and blood stages. It has long been a goal to understand the regulation of gene expression throughout each developmental stage. Previous attempts to apply functional genomics methods to address these questions used various approaches, including DNA microarrays (Hayward et al. 2000; Ben Mamoun et al. 2001; Le Roch et al. 2002), serial analysis of gene expression (Patankar et al. 2001), and mass spectrometry (Florens et al. 2002; Lasonder et al. 2002) on a limited number of samples from different developmental stages. While all of these approaches have provided insight into the biology of this organism, there have been no comprehensive analyses of any single developmental stage. Here we present an examination of the full transcriptome of one of these stages, the asexual intraerythrocytic developmental cycle (IDC), at a 1-h timescale resolution.

**Figure 1:**
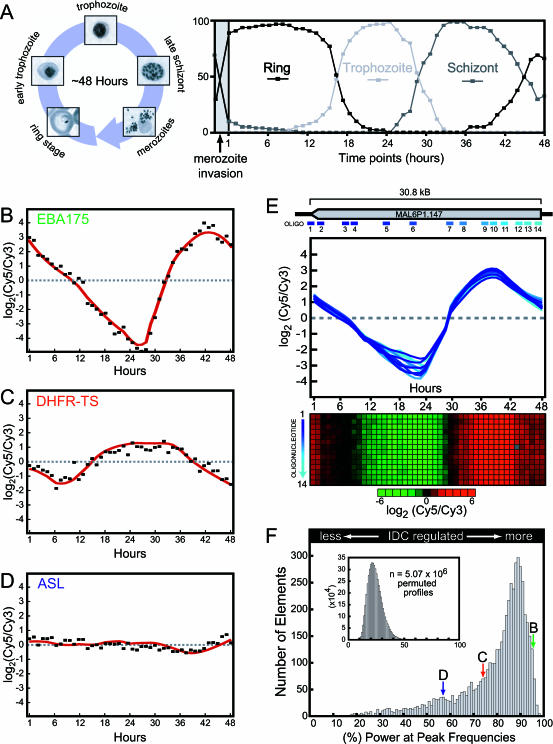
Parasite Culturing and Data Characteristics of the P. falciparum IDC Transcriptome Analysis (A) Giemsa stains of the major morphological stages throughout the IDC are shown with the percent representation of ring-, trophozoite-, or schizont-stage parasites at every timepoint. The 2-h invasion window during the initiation of the bioreactor culture is indicated (gray area). (B–D) Example expression profiles for three genes, encoding EBA175, DHFR-TS, and ASL, are shown with a loess fit of the data (red line). (E) MAL6P1.147, the largest predicted ORF in the *Plasmodium* genome, is represented by 14 unique DNA oligonucleotide elements. The location of each of the oligonucleotide elements within the predicted ORF and the corresponding individual expression profiles are indicated (oligo 1–14). A red/green colorimetric representation of the gene expression ratios for each oligonucleotide is shown below the graph. The pairwise Pearson correlation for these expression profiles is 0.98 ± 0.02. (F) The percentage of the power in the maximum frequency of the FFT power spectrum was used as an indicator of periodicity. A histogram of these values reveals a strong bias toward single-frequency expression profiles, indicating that the majority of P. falciparum genes are regulated in a simple periodic manner. This bias is eliminated when the percent power was recalculated using random permutations of the same dataset (inset). For reference, the locations of EBA175 (peak B), DHFR-TS (peak C), and ASL (peak D) are shown.

The 48-h P. falciparum IDC (Figure 1A) initiates with merozoite invasion of red blood cells (RBCs) and is followed by the formation of the parasitophorous vacuole (PV) during the ring stage. The parasite then enters a highly metabolic maturation phase, the trophozoite stage, prior to parasite replication. In the schizont stage, the cell prepares for reinvasion of new RBCs by replicating and dividing to form up to 32 new merozoites. The IDC represents all of the stages in the development of P. falciparum responsible for the symptoms of malaria and is also the target for the vast majority of antimalarial drugs and vaccine strategies.

Our laboratory has developed a *P. falciparum–*specific DNA microarray using long (70 nt) oligonucleotides as representative elements for predicted ORFs in the sequenced genome (strain 3D7) (Bozdech et al. 2003). Using this DNA microarray, we have examined expression profiles across 48 individual 1-h timepoints from the IDC of P. falciparum. Our data suggest that not only does P. falciparum express the vast majority of its genes during this lifecycle stage, but also that greater than 75% of these genes are activated only once during the IDC. For genes of known function, we note that this activation correlates well with the timing for the respective protein's biological function, thus illustrating an intimate relationship between transcriptional regulation and the developmental progression of this highly specialized parasitic organism. We also demonstrate the potential of this analysis to elucidate the function of the many unknown gene products as well as for further understanding the general biology of this parasitic organism.

## Results

### Expression Profiling of the IDC

The genome-wide transcriptome of the P. falciparum IDC was generated by measuring relative mRNA abundance levels in samples collected from a highly synchronized in vitro culture of parasites. The strain used was the well-characterized Honduran chloroquine-sensitive HB3 strain, which was used in the only two experimental crosses carried out thus far with P. falciparum (Walliker et al. 1987; Wellems et al. 1990). To obtain sufficient quantities of parasitized RBCs and to ensure the homogeneity of the samples, a large-scale culturing technique was developed using a 4.5 l bioreactor (see Materials and Methods). Samples were collected for a 48-h period beginning 1 h postinvasion (hpi). Culture synchronization was monitored every hour by Giemsa staining. We observed only the asexual form of the parasite in these stains. The culture was synchronous, with greater than 80% of the parasites invading fresh RBCs within 2 h prior to the harvesting of the first timepoint. Maintenance of synchrony throughout the IDC was demonstrated by sharp transitions between the ring-to-trophozoite and trophozoite-to-schizont stages at the 17- and 29-h timepoints, respectively (Figure 1A).

The DNA microarray used in this study consists of 7,462 individual 70mer oligonucleotides representing 4,488 of the 5,409 ORFs manually annotated by the malaria genome sequencing consortium (Bozdech et al. 2003). Of the 4,488 ORFs, 990 are represented by more than one oligonucleotide. Since our oligonucleotide design was based on partially assembled sequences periodically released by the sequencing consortium over the past several years, our set includes additional features representing 1,315 putative ORFs not part of the manually annotated collection. In this group, 394 oligonucleotides are no longer represented in the current assembled sequence. These latter ORFs likely fall into the gaps present in the published assembly available through the *Plasmodium* genome resource PlasmoDB.org (Gardner et al. 2002; Kissinger et al. 2002; Bahl et al. 2003).

To measure the relative abundance of mRNAs throughout the IDC, total RNA from each timepoint was compared to an arbitrary reference pool of total RNA from all timepoints in a standard two-color competitive hybridization (Eisen and Brown 1999). The transcriptional profile of each ORF is represented by the mean-centered series of ratio measurements for the corresponding oligonucleotide(s) (Figure 1B–1E). Inspection of the entire dataset revealed a striking nonstochastic periodicity in the majority of expression profiles. The relative abundance of these mRNAs continuously varies throughout the IDC and is marked by a single maximum and a single minimum, as observed for the representative schizont-specific gene, erythrocyte-binding antigen 175 (*eba175*), and the trophozoite-specific gene, dihydrofolate reductase–thymidylate synthetase (*dhfr-ts*) (Figure 1B and 1C). However, there is diversity in both the absolute magnitude of relative expression and in the timing of maximal expression (phase). In addition, a minority of genes, such as adenylosuccinate lyase (*asl*) (Figure 1D), displayed a relatively constant expression profile. The accuracy of measurements from individual oligonucleotides was further verified by the ORFs that are represented by more than one oligonucleotide feature on the microarray. The calculated average pairwise Pearson correlation (*r*) is greater than 0.90 for 68% (0.75 for 86%) of the transcripts represented by multiple oligonucleotides with detectable expression during the IDC (Table S1). Cases in which data from multiple oligonucleotides representing a single putative ORF disagree may represent incorrect annotation. The internal consistency of expression profile measurements for ORFs represented by more than one oligonucleotide sequence is graphically shown in Figure 1E for the hypothetical protein MAL6P1.147, the largest predicted ORF in the genome (31 kb), which is represented by 14 oligonucleotide elements spanning the entire length of the coding sequence. The average pairwise correlation (*r*) for these features is 0.98±0.02.

Periodicity in genome-wide gene expression datasets has been used to identify cell-cycle-regulated genes in both yeast and human cells (Spellman et al. 1998; Whitfield et al. 2002). Owing to the cyclical nature of the P. falciparum IDC dataset, a similar computational approach was taken. We performed simple Fourier analysis, which allowed us to calculate both the apparent phase and frequency of expression for each gene during the IDC (see Materials and Methods). The fast Fourier transform (FFT) maps a function in a time domain (the expression profile) into a frequency domain such that when the mapped function is plotted (the power spectra), sharp peaks appear at frequencies where there is intrinsic periodicity. The calculated power spectra for each expression profile confirmed the observation that the data are highly periodic. The majority of profiles exhibited an overall expression period of 0.75–1.5 cycles per 48 h.

We have used the FFT data for the purpose of filtering the expression profiles that are inherently noisy (i.e., that have low signal) or that lack differential expression throughout the IDC. Since the majority of the profiles display a single low-frequency peak in the power spectrum, we have taken advantage of this feature to classify profiles, similar to the application of a low-pass filter in signal processing. By measuring the power present in the peak frequency window (the main component plus two adjacent peaks) relative to the power present at all frequencies of the power spectrum, we were able to define a score (percent power) that we have used to stratify the dataset. The resulting distribution of expression profiles, scored in this way, is shown in Figure 1F for all oligonucleotides. For reference, the positions of profiles corresponding to *eba175* (peak B), *dhfr-ts* (peak C), and *asl* (peak D) are indicated. It is striking that 79.5% of the expression profiles have a very high score (greater than 70%). For comparison, we applied our FFT analysis to the Saccharomyces cerevisiae cell cycle data, yielding only 194 profiles (3.8%) above a 70% score (Figure S1). In addition, we randomly permuted the columns of the complete dataset 1,000 times, each time recalculating the FFT, for a total of 5 million profiles (see inset in Figure 1F). The randomized set exhibits essentially no periodicity: the probability of any random profile scoring above 70% is 1.3 × 10^−5^.

### P. falciparum Transcriptome Overview

**Figure 2:**
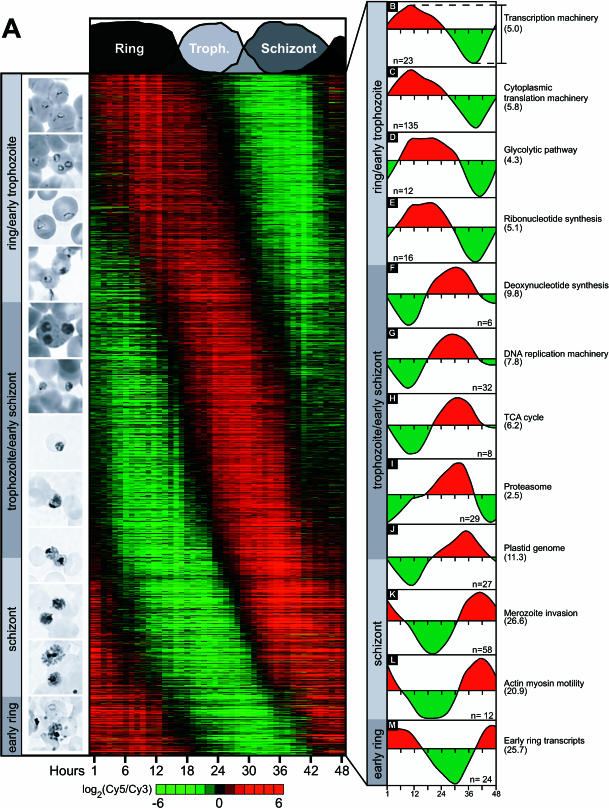
Overview of the P. falciparum IDC Transcriptome (A) A phaseogram of the IDC transcriptome was created by ordering the transcriptional profiles for 2,712 genes by phase of expression along the y-axis. The characteristic stages of intraerythrocytic parasite morphology are shown on the left, aligned with the corresponding phase of peak gene expression. (B–M) The temporal ordering of biochemical processes and functions is shown on the right. Each graph corresponds to the average expression profile for the genes in each set and the mean peak-to-trough amplitude is shown in parentheses.

To provide an overview of the IDC transcriptome, we selected all 3,719 microarray elements whose profiles exhibited greater than 70% of the power in the maximum frequency window and that were also in the top 75% of the maximum frequency magnitudes. Although hierarchical clustering is extremely useful for comparing any set of expression data, regardless of the experimental variables, we sought to specifically address temporal order within the dataset. To accomplish this, the FFT phase was used to order the expression profiles to create a phaseogram of the IDC transcriptome of P. falciparum (Figure 2A). The overview set represents 2,714 unique ORFs (3,395 oligonucleotides). An additional 324 oligonucleotides represent ORFs that are not currently part of the manually annotated collection.

The IDC phaseogram depicts a cascade of continuous expression lacking clear boundaries or sharp transitions. During the first half of the IDC, a large number of genes involved in general eukaryotic cellular functions are induced with broad expression profiles. This gradual continuum includes the transition from the ring to the early trophozoite stage and the trophozoite to the early schizont stage, encompassing approximately 950 and 1,050 genes, respectively. Next, the mid- and late-schizont stages are marked by a rapid, large amplitude induction of approximately 550 genes, many of which appear to be continually expressed into the early-ring stage. However, owing to the level of synchrony in the culture, the ring-stage signal may be partially attributed to cross-contamination from residual schizonts. In the final hours of the IDC, approximately 300 genes corresponding to the early-ring stage are induced, indicating that reinvasion occurs without obvious interruptions to initiate the next cycle. The expression profiles for developmentally regulated genes in the P. falciparum IDC transcriptome reveal an orderly timing of key cellular functions. As indicated in Figure 2B–2M, groups of functionally related genes share common expression profiles and demonstrate a programmed cascade of cellular processes that ensure the completion of the P. falciparum IDC.

### Ring and Early-Trophozoite Stage

In the following text, we have grouped the genes according to temporal expression phases based on their association with the common P. falciparum cytological stages.

Following invasion, approximately 950 ORFs are induced during the ring and early trophozoite stage, including genes associated with the cytoplasmic transcriptional and translational machinery, glycolysis and ribonucleotide biosynthesis (Figure 2B–2E). Represented in this group are 23 ORFs involved in transcription, including the four subunits of RNA polymerase I, nine subunits of RNA polymerase II, three subunits of RNA polymerase III, and four transcription factors. The average expression profile for this group is shown in Figure 2B. (See Table S2 for all functional group details.) Also in this set are three previously identified P. falciparum RNA polymerase genes: the large subunits of P. falciparum RNA polymerase I (Fox et al. 1993) and RNA polymerase II (Li et al. 1989) and RNA polymerase III (Li et al. 1991). The cytoplasmic translation gene group (Figure 2C) consists of 135 ORFs including homologues for 34 small and 40 large ribosomal subunits, 15 translation initiation factors, five translation elongation factors, 18 aminoacyl-tRNA synthetases, and 23 RNA helicases. In addition to the manually annotated ORFs, the translation gene group contains three ORFs predicted only by automated annotation including two ribosomal proteins (chr5.glm_215, chr5.glm_185) and a homologue of eIF-1A (chr11.glm_489) (PlasmoDB.org). In one case, chr5.glm_185 overlaps with the manually annotated ORF PFE0850w, which is found on the opposite strand. Oligonucleotide elements for both of these ORFs are present on the array. The oligonucleotide corresponding to the automated prediction yielded a robust FFT score and a phase consistent with the translation machinery, yet no PFE0850w expression was detected. These results suggest that the automated prediction for chr5.glm_185 most likely represents the correct gene model for this genomic locus and illustrates the use of the IDC expression data for further verification of the P. falciparum genome annotations.

Another set of 33 ORFs with homology to components of the translational machinery displayed an entirely distinct expression pattern, being induced during the late-trophozoite and early-schizont stage. This group includes 11 homologues of chloroplast ribosomal proteins, four mitochondrial/chloroplast elongation factors, and six amino acid tRNA synthetases (Table S2). These ORFs also share a common pattern of expression, suggesting that these factors are components of the mitochondrial and/or the plastid translation machinery. This observation is supported by the presence of predicted apicoplast-targeting signals in 18 of these proteins (PlasmoDB.org). In addition, one of these factors, ribosomal protein S9, has been experimentally immunolocalized within the plastid (Waller et al. 1998). These data suggest that the peak of expression for the cytoplasmic translation machinery occurs in the first half of the IDC, whereas plastid and mitochondrial protein synthesis is synchronized with the maturation of these organelles during the second half of the IDC.

In addition to transcription and translation, genes involved in several basic metabolic pathways were also induced during the ring and early-trophozoite stage, including glycolysis and ribonucleotide biosynthesis (Figure 2D and 2E). Unlike the majority of  P. falciparum biochemical processes, most of the enzymes involved in nucleotide metabolism and glycolysis have been identified (Reyes et al. 1982; Sherman 1998). The glycolysis group (Figure 2D) is tightly coregulated throughout the IDC and contains all of the 12 known enzymes. Expression initiates after reinvasion and continues to increase toward maximal expression during the trophozoite stage, when metabolism is at its peak. The glycolytic pathway is very well preserved in P. falciparum and exemplifies how data from this study can complement the homology-based interpretation of the genome. First, the genome contains two putative copies of pyruvate kinase on chromosomes 6 and 10, MAL6P1.160 and PF10_0363, respectively (Gardner et al. 2002). However, only one of these genes, MAL6P1.160, has a similar expression profile to the other known glycolytic enzymes, suggesting that this enzyme is the main factor of this step in the glycolytic pathway. Interestingly, PF10_0363 contains a putative apicoplast-targeting signal (PlasmoDB.org). In another case, the malaria genome sequencing consortium has predicted two homologues of triose phosphate isomerase, PF14_0378 and PFC0381w. The latter is not detected by our analysis, suggesting that this gene is utilized in another developmental stage or may be a nonfunctional, redundant homologue.

P. falciparum parasites generate pyrimidines through a de novo synthesis pathway while purines must be acquired by the organism through a salvage pathway (Gero and O'Sullivan 1990). The mRNA levels of 16 enzymes corresponding to members of the pyrimidine ribonucleotide synthesis pathway, beginning with carbamoyl phosphate synthetase and ending with CTP synthetase, were uniformly induced immediately after invasion (Figure 2E). The relative abundance of these transcripts peaked at approximately 18–22 hpi and then rapidly declined. Similar expression characteristics were detected for the enzymes of the purine salvage pathway, including the nucleoside conversion enzymes, hypoxanthine–guanine–xanthine phosphoribosyltransferase, and both guanylate and adenylate kinases (Figure 2E; Table S2).

### Trophozoite and Early-Schizont Stage

The mRNA expression data indicate that ribonucleotide and deoxyribonucleotide production is clearly bifurcated into two distinct temporal classes. While ribonucleotide synthesis is required in the early stages of the IDC, deoxyribonucleotide metabolism is a trophozoite/early-schizont function. mRNA transcripts for enzymes that convert ribonucleotides into deoxyribonucleotides, including DHFR-TS and both subunits of ribonucleotide reductase, were induced approximately at 10 hpi, peaking at approximately 32 hpi (Figure 2F). This represents a temporal shift from the induction of ribonucleotide synthesis of approximately 8–10 h. The expression of the deoxyribonucleotide biosynthesis is concomitant with the induction of DNA replication machinery transcripts, reflecting a tight relationship between DNA synthesis and production of precursors for this process.

Thirty-two ORFs with homologies to various eukaryotic DNA replication machinery components are transcribed during the late-trophozoite and early-schizont stage. The timing of their transcription presages cell division. This functional gene group (Figure 2G), with peak expression around 32 hpi, contains the previously characterized P. falciparum DNA Polα, DNA Polδ, and proliferating cell nuclear antigen, as well as the vast majority of the DNA replication components predicted by the malaria genome sequencing consortium (Gardner et al. 2002). These additional components include eight predicted DNA polymerase subunits, two putative origin recognition complex subunits, six minichromosome maintenance proteins, seven endo- and exonucleases, seven replication factor subunits, and two topoiosomerases. Interestingly, a number of proteins typically required for eukaryotic DNA replication, including the majority of the subunits of the origin recognition complex, have not yet been identified by conventional sequence similarity searches of the P. falciparum genome.

All genes necessary for the completion of the tricarboxylic acid (TCA) cycle were detected in the *Plasmodium* genome (Gardner et al. 2002), although earlier studies indicate an unconventional function for this metabolic cycle. These studies suggest that the TCA cycle does not play a major role in the oxidation of glycolytic products. Instead, it is essential for the production of several metabolic intermediates, such as succinyl-CoA, a precursor of porphyrin biosynthesis (Sherman 1998). The peak of expression for all TCA factors was detected during the late-trophozoite and early-schizont stage (Figure 2H). Consistent with the model suggesting a disconnection of the TCA cycle from glycolysis during the IDC, no expression was detected for the subunits of the pyruvate dehydrogenase complex, including the α and β chains of pyruvate dehydrogenase and dihydrolipoamide S-acetyl transferase, the typical links between glycolysis and the TCA cycle. On the other hand, expression of TCA cycle genes is well synchronized with the expression of a large number of mitochondrial genes, including the three ORFs of the mitochondrial genome (Feagin et al. 1991), and several factors of electron transport (Table S2). Although some of the TCA cycle proteins have been localized to the cytoplasm (Lang-Unnasch 1992), the expression data suggest an association of this biochemical process with mitochondrial development and possibly with the abbreviated electron transport pathway detected in this organelle.

### Schizont Stage

A transition from early to mid-schizont is marked by the maximal induction of 29 ORFs predicted to encode various subunits of the proteasome (Figure 2I). Seven α and six β subunits of the 20S particle and 16 ORFs of the 19S regulatory particle were identified in this gene group. The common expression profile for the subunits of both of the 26S particle complexes suggests the involvement of ubiquitin-dependent protein degradation in the developmental progression of the parasite. The peak of proteasome expression coincides with a transition in the IDC transcriptome from metabolic and generic cellular machinery to specialized parasitic functions in the mid-schizont stage. This suggests an association between transcriptional regulation and protein turnover during this and possibly other transitions during the progression of the P. falciparum IDC.

**Figure 3:**
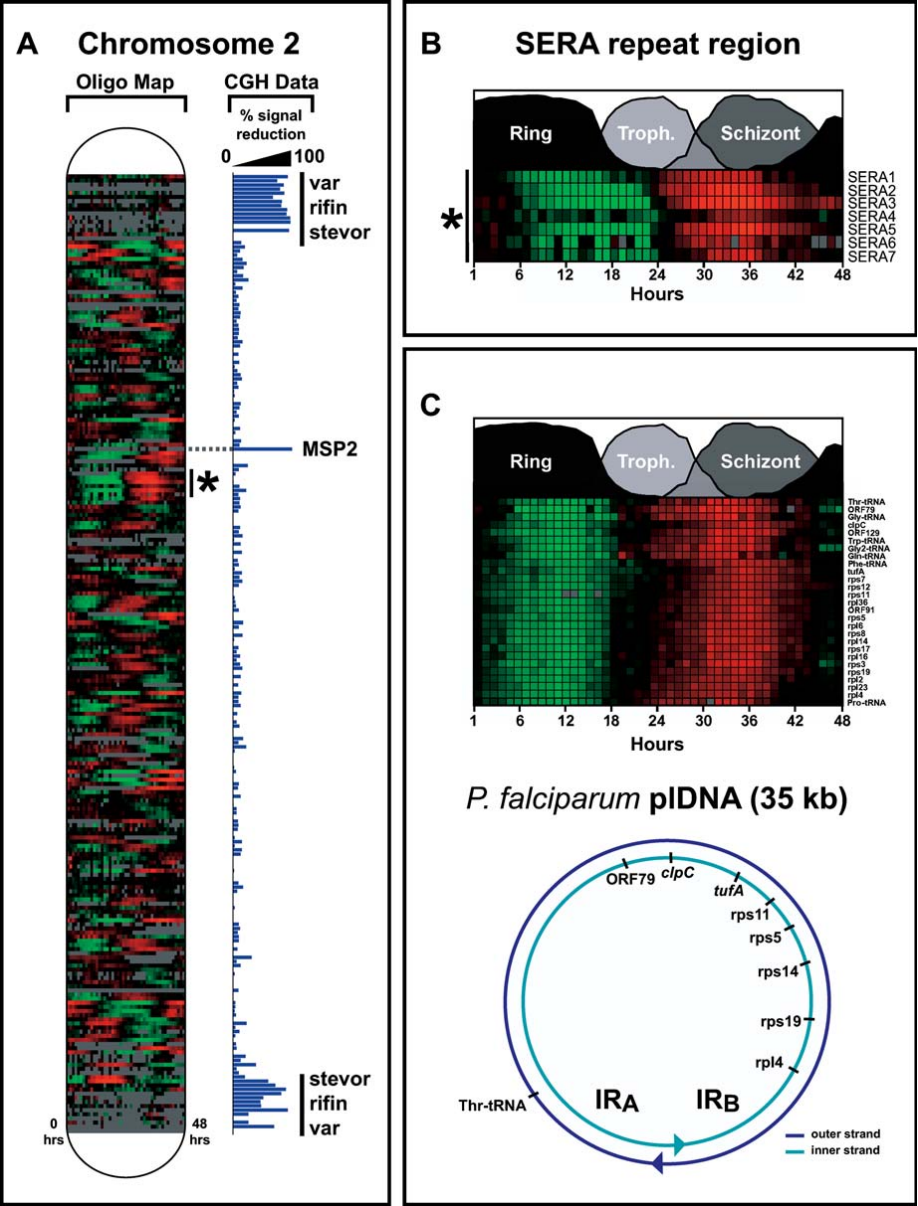
Coregulation of Gene Expression along the Chromosomes of P. falciparum Is Rare, While Plastid Gene Expression Is Highly Coordinated Expression profiles for oligonucleotides are shown as a function of location for Chromosome 2 ([A], Oligo Map). With the exception of the SERA locus (B), coregulated clusters of adjacent ORFs are seldom observed, indicating that expression phase is largely independent of chromosomal position. (C) In contrast to the nuclear chromosomes, the polycistronic expression of the circular plastid genome is reflected in the tight coregulation of gene expression. This is an expanded view of the plastid-encoded genes from Figure 2J. Genomic differences between strain 3D7, from which the complete genome was sequenced, and strain HB3 were measured by CGH. The relative hybridization between the gDNA derived from these two strains is shown as a percent reduction of the signal intensity for 3D7 ([A],**** CGH Data). Differences between the two strains are predominately located in the subtelomeric regions that contain the highly polymorphic *var, rifin,* and *stevor* gene families. Intrachromosomal variations, as observed for the *msp2* gene, were rare.

In the schizont stage, one of the first specialized processes induced was expression from the plastid genome (Figure 2J). The essential extrachromosomal plastid (or apicoplast) genome contains 60 potentially expressed sequences, including ribosomal proteins, RNA polymerase subunits, ribosomal RNAs, tRNAs, and nine putative ORFs, including a ClpC homologue (Wilson et al. 1996). Very little is known about the regulation of gene expression in the plastid, but it is thought to be polycistronic (Wilson et al. 1996). In support of this observation, we find that 27 of the 41 plastid-specific elements present on our microarray displayed an identical expression pattern (Figure 3C). The remaining elements correspond mainly to tRNAs and failed to detect appreciable signal. The highly coordinated expression of the plastid genome, whose gene products are maximally expressed in the late-schizont stage, is concomitant with the replicative stage of the plastid (Williamson et al. 2002). Note that not all plastid ORFs are represented on the microarray used in this study, and thus it is a formal possibility that the expression of the missing genes may differ from those shown in Figure 3C.

Offset from the plastid by approximately 6 h, a set of approximately 500 ORFs exhibited peak expression during the late-schizont stage. Merozoite invasion of a new host cell is a complex process during which the parasite must recognize and dock onto the surface of the target erythrocyte, reorient with its apical tip toward the host cell, and internalize itself through invagination of the erythrocytic plasma membrane. The entire sequence of invasion events is facilitated by multiple receptor–ligand interactions with highly specialized plasmodial antigens (Cowman et al. 2000). The merozoite invasion group contains 58 ORFs, including 26 ORFs encoding antigens previously demonstrated to be important for the invasion process (see Figure 2K). These include integral membrane proteins delivered to the merozoite surface from the micronemes (AMA1 and EBA175), GPI-anchored proteins of the merozoite membrane (MSP1, MSP4, and MSP5), proteins extrinsically associated with the merozoite surface during their maturation in the PV (MSP3 and MSP6), and soluble proteins secreted to the parasite–host cell interface (RAP1, RAP2, and RAP3). In addition, late-schizont-specific expression was observed for several antigens whose functions are not completely understood, but which have been associated with the invasion process. These ORFs include the merozoite-capping protein (MCP1), erythrocyte-binding-like protein 1 (EBL1), reticulocyte-binding proteins (RBP1 and RBP2), acid basic repeat antigen (ABRA), MSP7, and a homologue of the Plasmodium yoelii merozoite antigen 1. As expected, peak expression of these antigens coincides with the maturation of merozoites and development of several apical organelles, including rhoptries, micronemes, and dense granules. Many of these proteins have been considered as vaccine candidates since antibodies against these antigens were readily detected in the immune sera of both convalescent patients as well as individuals with naturally acquired immunity (Preiser et al. 2000).

The sensitivity of invasion to protease and kinase inhibitors indicates an essential role for these activities in merozoite release as well as in the reinvasion process (Dluzewski and Garcia 1996; Blackman 2000; Greenbaum et al. 2002). The merozoite invasion gene group contains three serine proteases, including PfSUB1, PfSUB2, and an additional homologue to plasmodial subtilases (PFE0355c), and two aspartyl proteases, plasmepsin (PM) IX and X. Peak expression during the mid-schizont stage was also observed for seven members of the serine repeat antigen (SERA) family, all of which contain putative cysteine protease domains. In addition to the proteases, expression of 12 serine/threonine protein kinases and three phophorylases was tightly synchronized with the genes of the invasion pathway, including six homologues of protein kinase C, three Ca^+^-dependent and two cAMP-dependent kinases, phosphatases 2A and 2B, and protein phosphatase J.

Another functionally related gene group whose expression is sharply induced during the late-schizont stage includes components of actin–myosin motors (see Figure 2L) (Pinder et al. 2000). As in other apicomplexa, actin and myosin have been implicated in host cell invasion (Opitz and Soldati 2002). Schizont-specific expression was observed for three previously described class XIV myosin genes, one associated light chain, two actin homologues, and three additional actin cytoskeletal proteins, including actin-depolymerizing factor/cofilin (two isoforms) and coronin (one isoform). Although the molecular details of plasmodial actin–myosin invasion are not completely understood, the tight transcriptional coregulation of the identified factors indicates that the examination of schizont-specific expression may help to identify additional, possibly unique elements of this pathway.

### Early-Ring Stage

The expression data are continuous throughout the invasion process, with no observable abrupt change in the expression program upon successful reinvasion. However, a set of approximately 300 ORFs whose expression is initiated in the late-schizont stage persists throughout the invasion process and peaks during the early-ring stages (see Figure 2M). It was previously determined that immediately after invasion, a second round of exocytosis is triggered, ensuring successful establishment of the parasite within the host cell (Foley et al. 1991). One of the main P. falciparum virulence factors associated with this process is ring-infected surface antigen 1 (RESA1). RESA1 is secreted into the host cell cytoplasm at the final stages of the invasion process, where it binds to erythrocytic spectrin, possibly via its DnaJ-like chaperone domain (Foley et al. 1991). The early stages of the IDC contain a variety of putative molecular chaperones in addition to RESA1, including RESA2 and RESAH3, plus five additional proteins carrying DnaJ-like domains. However, the functional roles of these chaperones remain unclear. Despite the cytoplasmic role of RESA1, abundant antibodies specific for RESA1 are present in individuals infected with P. falciparum, indicating that RESA1 is also presented to the host immune system (Troye-Blomberg et al. 1989). Several genes encoding additional antigenic factors are found among the early ring gene group, including frequently interspersed repeat antigen (FIRA), octapeptide antigen, MSP8, and sporozoite threonine- and asparagine-rich protein (STARP). Like RESA1, antibodies against these antigens are also found in the sera of infected individuals, suggesting that the final stages of invasion might be a target of the immune response.

Overall, the genes expressed during the mid- to late-schizont and early-ring stage encode proteins predominantly involved in highly parasite-specific functions facilitating various steps of host cell invasion. The expression profiles of these genes are unique in the IDC because of the large amplitudes and narrow peak widths observed. The sharp induction of a number of parasite-specific functions implies that they are crucial for parasite survival in the mammalian host and hence should serve as excellent targets for both chemotherapeutic and vaccine-based antimalarial strategies.

### IDC Transcriptional Regulation and Chromosomal Structure

Transcriptional regulation of chromosomal gene expression in P. falciparum is thought to be monocistronic, with transcriptional control of gene expression occurring through regulatory sequence elements upstream and downstream of the coding sequence (Horrocks et al. 1998). This is in contrast to several other parasites, such as Leishmania sp., in which polycistronic mRNA is synthesized from large arrays of coding sequences positioned unidirectionally along the arms of relatively short chromosomes (Myler et al. 2001). Recent proteomic analyses failed to detect any continuous chromosomal regions with common stage-specific gene expression in several stages of the P. falciparum lifecycle (Florens et al. 2002). However, transcriptional domains have previously been suggested for Chromosome 2 (Le Roch et al. 2002). The availability of the complete P. falciparum genome coupled with the IDC transcriptome allows us to investigate the possibility of chromosomal clustering of gene expression (see Figure 3A). To systematically explore the possibility of coregulated expression as a function of chromosomal location, we applied a Pearson correlation to identify similarities in expression profiles among adjacent ORFs. The pairwise Pearson correlation was calculated for every ORF pair within each chromosome (Figure S2). Gene groups in which the correlation of 70% of the possible pairs was greater than *r* = 0.75 were classified as putative transcriptionally coregulated groups. Using these criteria, we identified only 14 coregulation groups consisting of greater than three genes, with the total number of genes being 60 (1.4% of all represented genes) (Table S3). In eight of the 14 groups, the coregulation of a pair of genes may be explained by the fact that they are divergently transcribed from the same promoter. A set of 1,000 randomized permutations of the dataset yielded 2.25 gene groups. Contrary to the nuclear chromosomes, there was a high correlation of gene expression along the plastid DNA element, consistent with polycistronic transcription (see Figure 3C). The average pairwise Pearson correlation for a sliding window of seven ORFs along the plastid genome is 0.92±0.03.

The largest group demonstrating coregulation on the nuclear chromosomes corresponds to seven genes of the SERA family found on Chromosome 2 (see Figure 3B) (Miller et al. 2002). Besides the SERA gene cluster and a group containing three ribosomal protein genes, no additional functional relationship was found among the other chromosomally adjacent, transcriptionally coregulated gene groups. The limited grouping of regional chromosomal expression was independent of strand specificity and, with the exception of the SERA group, did not overlap with the groups of “recently duplicated genes” proposed by the malaria genome sequencing consortium (Gardner et al. 2002).

Three major surface antigens, the *var*, *rifin*, and *stevor* families, have a high degree of genomic variability and are highly polymorphic between strains and even within a single strain (Cheng et al. 1998; Afonso Nogueira et al. 2002; Gardner et al. 2002). Expression profiles for only a small subset of these genes were detected in the IDC transcriptome and were typically characterized by low-amplitude profiles. This could be due to two nonmutually exclusive possibilities: first, the HB3 DNA sequence for these genes may be substantially rearranged or completely deleted relative to the reference strain, 3D7; second, only a few of these genes may be selectively expressed, as has been proposed (Deitsch et al. 2001). To identify regions of genomic variability between 3D7 and HB3, we performed microarray-based comparative genomic hybridization (CGH) analysis. Array-based CGH has been performed with human cDNA and bacterial artificial chromosome-based microarrays to characterize DNA copy-number changes associated with tumorigenesis (Gray and Collins 2000; Pollack et al. 2002). Using a similar protocol, CGH analysis revealed that the majority of genetic variation between HB3 and 3D7 is confined to the subtelomeric chromosomal regions containing the aforementioned gene families (Figure 3A; Figure S3). Only 28.3% of *rifin*, 47.1% of *var*, and 51.0% of *stevor* genes predicted for the 3D7 strain were detected for the HB3 genomic DNA (gDNA) when hybridized to the 3D7-based microarray. Thus, the underrepresentation of these gene families in the HB3 IDC transcriptome is likely due to the high degree of sequence variation present in these genes. Excluding the three surface antigen families in the subtelomeric regions, 97% of the remaining oligonucleotide microarray elements exhibit an equivalent signal in the CGH analysis. However, 144 of the differences detected by CGH reside in internal chromosomal regions and include several previously identified plasmodial antigens: MSP1, MSP2 (Figure 3A), S antigen, EBL1, cytoadherence-linked asexual gene 3.1 (CLAG3.1), glutamine-rich protein (GLURP), erythrocyte membrane protein 3 (PfEMP3), knob-associated histidine-rich protein (KAHRP), and gametocyte-specific antigen Pfg377 (Table S4). These results demonstrate a high degree of genetic variation within the genes considered to be crucial for antigenic variation between these two commonly used laboratory strains of P. falciparum.

### Implications for Drug Discovery

The majority of the nuclear-encoded proteins targeted to the plastid are of prokaryotic origin, making them excellent drug targets (McFadden and Roos 1999). Moreover, inhibitors of plastid-associated isoprenoid biosynthesis, DNA replication, and translation have been shown to kill the P. falciparum parasite, demonstrating that the plastid is an essential organelle (Fichera and Roos 1997; Jomaa et al. 1999). The plastid has been implicated in various metabolic functions, including fatty acid metabolism, heme biosynthesis, isoprenoid biosynthesis, and iron–sulfur cluster formation (Wilson 2002). It is clear that, within the plastid, functional ribosomes are assembled to express the ORFs encoded by the plastid genome (Roy et al. 1999). However, nuclear-encoded components are required to complete the translational machinery as well as for all other plastid metabolic functions. A bipartite signal sequence is required for efficient transport of these nuclear proteins from the cytoplasm to the plastid via the endoplasmic reticulum (Waller et al. 2000). Computational predictions suggest that the P. falciparum genome may contain over 550 nuclear-encoded proteins with putative transit peptides (Zuegge et al. 2001; Foth et al. 2003).

**Figure 4:**
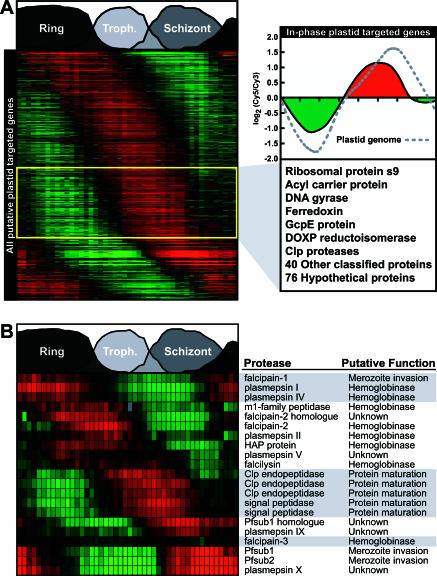
Temporal Distribution of the Apicoplast-Targeted Proteins and P. falciparum Proteases, Potential Antimalarial Drug Candidates (A) The expression profiles of all putative plastid-targeted genes represented on our microarray are shown. The yellow box encompasses a highly synchronized group of genes, which are in-phase with plastid genome expression. The average expression profile for this in-phase group of genes is shown and includes most of the known apicoplast-targeted genes as well as many hypothetical genes. For reference, the average expression profile for the plastid genome is shown (dashed gray line). (B) Proteases represent an attractive target for chemotherapeutic development. The broad range of temporal expression for various classes of proteases and their putative functions are displayed. Abbreviations: HAP, histo-aspartyl protease (PM III); Clp, caseineolytic protease; sub1, 2, subtilisin-like protease 1 and 2.

Given that over 10% of the ORFs in the P. falciparum genome are predicted to contain an apicoplast-targeting sequence, we sought to use the IDC transcriptome as a means to narrow the search space for candidate apicoplast-targeted genes. As mentioned above, the expression profiles for genes encoded on the plastid genome are tightly coordinated (see Figure 3C). We reasoned that genes targeted to the plastid would be expressed slightly before or coincidentally with the plastid genome. Therefore, we utilized the FFT phase information to identify ORFs in phase with expression of the plastid genome (see Materials and Methods) (Table S5). Because the genes of the plastid genome are maximally expressed between 33 and 36 hpi, we searched for all genes in the dataset with an FFT phase in this time window and then cross-referenced the list of predicted apicoplast-targeted sequences (PlasmoDB.org), resulting in a list of 124 in-phase apicoplast genes (Figure 4A). Within this list are two ORFs that have been directly visualized in the apicoplast, acyl carrier protein and the ribosomal subunit S9 (Waller et al. 1998), as well as many ORFs associated with the putative plastid ribosomal machinery, enzymes involved in the nonmevalonate pathway, additional caseineolytic proteases (Clps), the reductant ferredoxin, and replication/transcriptional machinery components. However, this list contains only 14 of the 43 proteins categorized in the Gene Ontology (GO) assignments at PlasmoDB.org as apicoplast proteins by inference from direct assay (IDA). In addition, 30% of the nuclear-encoded translational genes that are not coexpressed with the known cytoplasmic machinery are found within this small group of genes. More importantly, 76 ORFs (62%) are of unknown function, with little or no homology to other genes. This limited subgroup of putative plastid-targeted ORFs are likely excellent candidates for further studies in the ongoing search for malaria-specific functions as putative drug targets.

Similarly, P. falciparum proteases have received much attention, since they are candidates as drug targets and have been shown to play important roles in regulation as well as metabolism throughout the IDC (Rosenthal 2002). A temporal ordering of expression profiles for several well-characterized P. falciparum proteases is shown in Figure 4B, demonstrating the broad significance of these enzymes throughout the IDC. One of the principal proteolytic functions is considered to be the degradation of host cell hemoglobin in the food vacuole (FV) to produce amino acids essential for protein synthesis. This elaborate process is carried out by a series of aspartyl proteases, cysteine proteases, metalloproteases, and aminopeptidases (Francis et al. 1997).

A family of ten aspartyl proteases, the plasmepsins (PMs), has been identified in the P. falciparum genome, four of which have been characterized as bona fide hemoglobinases: PM I, II, III (a histo-aspartic protease [HAP]), and IV (Coombs et al. 2001). Our data reveal that the PMs are expressed at different times throughout the lifecycle, suggesting that they are involved in different processes throughout the IDC. PM I, II, HAP, and PM IV are adjacent to one another on Chromosome 14 and have been localized to the FV. While HAP and PM II are expressed in the mid-trophozoite stage, during peak hemoglobin catabolism, PMI and IV are maximally expressed in the ring stage along with the cysteine protease falcipain-1 (FP-1). FP-1 has recently been implicated in merozoite invasion and has been localized to the interior of the PV (Greenbaum et al. 2002). The coincident expression of these proteases implies that the development of the PV and the FV occurs during the very early-ring stage. This observation is corroborated by similar expression profiles for the PV-associated protein RESA1 and the FV protein PGH1. Subsequently, a second group of hemoglobinases, including the m1-family aminopeptidase, FP-2, and falcilysin, is expressed simultaneously with HAP and PM II during the trophozoite stage of the IDC. The expression of PM V and the newly identified FP- 2 homologue during this stage suggests they are also important in the trophozoite stage. The other known falcipain, FP-3, does not show a marked induction in expression throughout the IDC. We fail to detect any transcripts for PM VI, VII, and VIII during the IDC. These genes may have roles in any of the other sexual, liver, or mosquito stages of development.

In addition to the hemoglobinases, P. falciparum contains a variety of proteases involved in cellular processing, including a group of Clps and signal peptidases that are all expressed maximally at the late-trophozoite stage (Figure 4B). The timing of these genes may play a key role in protein maturation during trafficking to various compartments, including the plastid. The three Clps contain putative leader peptides and may actually function within the plastid. Finally, a group of proteases are expressed in the schizont stage and include the P. falciparum subtilisin-like proteases PfSUB1 and PfSUB2 as well as PMs IX and X. PfSUB1 and PfSUB2 are believed to be involved in merozoite invasion and have been localized apically in the dense granules. Interestingly, there are two PfSUB1 protease homologues (PFE0355c and PFE0370c); PM X parallels the expression of PfSUB1 (PFE0370c), suggesting that aspartyl proteases may also be involved in merozoite invasion. In addition, the phase of the PfSUB1 homologue suggests a concomitant role, with PM IX slightly preceding merozoite invasion. In total, we have detected gene expression for over 80 putative proteases throughout the entire IDC (Table S6). This set includes over 65 proteases from a group of recently predicted proteases (Wu et al. 2003). The differing temporal expression of these proteases may allow for a multifaceted approach toward identifying protease inhibitors with efficacy at all stages of the IDC.

### Implications for New Vaccine Therapies

Merozoite invasion is one of the most promising target areas for antimalarial vaccine development (Good 2001). Many vaccine efforts thus far have focused primarily on a set of plasmodial antigens that facilitate receptor–ligand interaction between the parasite and the host cell during the invasion process (Preiser et al. 2000) (see Figure 2K and 2M). Merozoite invasion antigens are contributing factors to naturally acquired immunity, triggering both humoral and antibody-independent cell-mediated responses (Good and Doolan 1999). Antibodies against these antigens have been demonstrated to effectively block the merozoite invasion process in vitro and in animal models (Ramasamy et al. 2001). Owing to the highly unique character of merozoite surface antigens, homology-based searches have yielded only a limited set of additional invasion factors.

**Figure 5:**
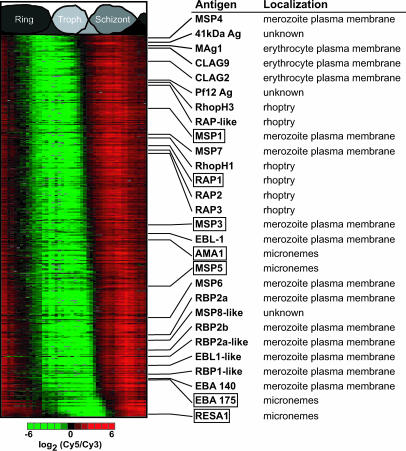
Phaseogram of Putative Vaccine Targets The similarity of all expression profiles to seven known vaccine candidates (boxed) was calculated. The top 5% of similar profiles correspond to 262 ORFs, 28 of which have been previously associated with plasmodial antigenicity and the process of merozoite invasion.

We utilized the IDC transcriptome to predict a set of likely invasion proteins by identifying expression profiles with characteristics similar to previously studied merozoite invasion proteins. The expression profiles for all known invasion factors undergo a sharp induction during the mid- to late-schizont stage and are characterized by large expression amplitudes (see Figure 2A). Among these proteins are seven of the best-known malaria vaccine candidates, including AMA1, MSP1, MSP3, MSP5, EBA175, RAP1, and RESA1. To identify ORFs with a possible involvement in the merozoite invasion process, we have calculated the similarity, by Euclidian distance, between the expression profiles of these seven vaccine candidates and the rest of the IDC transcriptome. A histogram of the distance values reveals a bimodal distribution with 262 ORFs in the first peak of the distribution (Figure S4). This represents the top 5% of expression profiles when ranked by increasing Euclidian distance (Table S7). In addition to the seven vaccine candidate genes used for the search, essentially all predicted P. falciparum merozoite-associated antigens were identified in this gene set (Figure 5). These include the GPI-anchored MSP4; several integral merozoite membrane proteins, such as EBA140 and EBL1; three RBPs (RBP1, RBP2a, RBP2b); and a previously unknown RBP homologue. In addition, components of two proteins secreted from the rhoptries to the host cell membranes, RhopH1 and RhopH3, or to the PVs RAP1, RAP2, and RAP3 were found in the selected set. Surprisingly, CLAG2 and CLAG9 were also classified into the merozoite invasion group. Although the biological function of these genes is believed to be associated with cytoadherence of the infected erythrocyte to the vascular endothelium, a highly related homologue, CLAG3.1 (RhopH1), was recently detected in the rhoptries, suggesting a possible secondary role for these genes in merozoites (Kaneko et al. 2001).

A number of antigens are presently in various stages of clinical trials and are yielding encouraging results (Good et al. 1998). However, many single-antigen vaccine studies indicate that the most promising approach will require a combination of antigenic determinants from multiple stages of the complex plasmodial lifecycle (Kumar et al. 2002). Searches for new target antigens in the P. falciparum genome are thus vital to the development of future vaccines, since no fully protective vaccine has been assembled thus far. Of the 262 ORFs whose expression profiles were closest to the profiles of the seven major vaccine candidates, 189 are of unknown function. These ORFs represent a candidate list for new vaccine targets.

## Discussion

The transcriptome of the IDC of P. falciparum constitutes an essential tool and baseline foundation for the analysis of all future gene expression studies in this organism, including response to drugs, growth conditions, environmental perturbations, and genetic alterations. Essentially all experiments involving asexual intraerythrocytic-stage parasites must be interpreted within the context of the ongoing cascade of IDC-regulated genes.

In our global analysis of the P. falciparum transcriptome, over 80% of the ORFs revealed changes in transcript abundance during the maturation of the parasite within RBCs. The P. falciparum IDC significantly differs from the cell cycles of the yeast S. cerevisiae (Spellman et al. 1998) and human HeLa (Whitfield et al. 2002) cells, during which only 15% of the total genome is periodically regulated. Instead, the P. falciparum IDC resembles the transcriptome of the early stages of Drosophila melanogaster development, which incorporates the expression of over 80% of its genome as well (Arbeitman et al. 2002). Unlike the development of multicellular eukaryotes, there is no terminal differentiation and, with the exception of gametocytogenesis, the parasite is locked into a repeating cycle. In this respect, the P. falciparum IDC mirrors a viral-like lifecycle, in which a relatively rigid program of transcriptional regulation governs the progress of the course of infection.

The lack of continuous chromosomal domains with common expression characteristics suggests that the genes are regulated individually, presumably via distinct sets of *cis*- and *trans*-acting elements. However, the extent and the simple mechanical character of transcriptional control observed in the IDC suggest a fundamentally different mode of regulation than what has been observed in other eukaryotes. It is plausible that a comparatively small number of transcription factors with overlapping binding site specificities could account for the entire cascade. While further experiments are ongoing, it may be the case that P. falciparum gene regulation is streamlined to the extent that it has lost the degree of dynamic flexibility observed in other unicellular organisms, from Escherichia coli to yeast. This observation also implies that disruption of a key transcriptional regulator, as opposed to a metabolic process, may have profound inhibitory properties. While a few putative transcription factors have been identified in the P. falciparum genome, no specific regulatory elements have been defined in basepair-level detail. A further analysis of the upstream regions of genes with similar phases should facilitate the elucidation of regulatory regions and their corresponding regulatory proteins.

In general, the timing of mRNA expression for a given gene during the IDC correlates well with the function of the resultant protein. For example, replication of the genome occurs in the early-schizont stage and correlates well with the peak expression of all factors of DNA replication and DNA synthesis. Also, organellar biogenesis of several intracellular compartments such as mitochondria, the plastid, or the apical invasion organelles is concomitant with the maximal induction of mRNAs encoding proteins specific to these organelles. In addition, our data are generally in good agreement with proteomic analyses that have detected intraerythrocytic-stage proteins from the merozoite, trophozoite, and schizont stages. More than 85% of the 1,588 proteins detected in these studies were also expressed in our analysis (Florens et al. 2002; Lasonder et al. 2002). However, a more detailed proteomic analysis at different stages of the IDC will be needed to ascertain the temporal changes of these proteins.

We initially expected that a high percentage of the genome would be specialized for each lifecycle stage (mosquito, liver, blood), yet this was not observed; the mRNA transcripts for 75% of proteins determined to be gamete-, gametocyte-, or sporozoite-specific by mass spectrometry are also transcribed in the plasmodial IDC. These findings confirm previous studies demonstrating that not only genes used for generic cellular processes are present in multiple developmental stages, but also factors of highly specialized *Plasmodium* functions (Gruner et al. 2001). This may indicate that only a small portion of the genome may actually be truly specific to a particular developmental stage and that the majority of the genome is utilized throughout the full lifecycle of this parasite. It is also feasible to speculate that a multilayer regulatory network is employed in the progression of the entire P. falciparum lifecycle. In this model, the same *cis*- and *trans*-acting regulatory elements driving the actual mRNA production in IDC are utilized in other developmental stages. These elements are then controlled by an alternate subset of factors determining the status of the lifecycle progression.

These findings also outline two contrasting properties of the P. falciparum genome. The *Plasmodium* parasite devotes 3.9% of its genome to a complex system of antigenic determinants essential for host immune evasion during a single developmental stage (Gardner et al. 2002). On the other hand, large portions of the genome encode proteins used in multiple stages of the entire lifecycle. Such broad-scope proteins might be excellent targets for both vaccine and chemotherapeutic antimalarial strategies, since they would target several developmental stages simultaneously. While there are certainly proteins specific to these nonerythrocytic stages, a complementary analysis of both proteomic and genomic datasets will facilitate the search.

With malaria continuing to be a major worldwide disease, advances toward understanding the basic biology of P. falciparum remain essential. Our analysis of the IDC transcriptome provides a first step toward a comprehensive functional analysis of the genome of P. falciparum. The genome-wide transcriptome will be useful not only for the further annotation of many uncharacterized genes, but also for defining the biological processes utilized by this highly specialized parasitic organism. Importantly, candidate groups of genes can be identified that are both functionally and transcriptionally related and thus provide focused starting points for the further elucidation of genetic and mechanistic aspects of P. falciparum. Such biological characterizations are presently a major objective in the search for novel antimalarial strategies. The public availability of the dataset presented in this study is intended to provide a resource for the entire research community to extend the exploration of P. falciparum beyond the scope of this publication. All data will be freely accessible at two sites: http://plasmodb.org and http://malaria.ucsf.edu.

## Materials and Methods

### 

#### Cell culture.

A large-scale culture of P. falciparum (HB3 strain) was grown in a standard 4.5 l microbial bioreactor (Aplikon, Brauwweg, Netherlands) equipped with a Bio Controller unit ADI 1030 (Aplikon, Brauwweg, Netherlands). Cells were initially grown in a 2% suspension of purified human RBCs and RPMI 1640 media supplemented with 0.25% Albumax II (GIBCO, Life Technologies, San Diego, California, United States), 2 g/l sodium bicarbonate, 0.1 mM hypoxanthine, 25 mM HEPES (pH 7.4), and 50 μg/l gentamycin, at 37°C, 5% O_2_, and 6% CO_2_. Cells were synchronized by two consecutive sorbitol treatments for three generations, for a total of six treatments. Large-scale cultures contained 32.5 mM HEPES (pH 7.4). The bioreactor culture was initiated by mixing 25.0 ml of parasitized RBCs (20% late schizonts, approximately 45 hpi) with an additional 115.0 ml of purified RBC in a total of 1.0 l of media (14% hematocrit). Invasion of fresh RBCs occurred during the next 2 h, raising the total parasitemia from an initial 5% to 16%. After this period, the volume of the culture was adjusted to 4.5 l, bringing the final RBC concentration to approximately 3.3% to reduce the invasion of remaining cells. Immediately after the invasion period, greater than 80% of the parasites were in the ring stage. Temperature and gas conditions were managed by the Bio Controller unit. Over the course of 48 h, 3–4 ml of parasitized RBCs was collected every hour, washed with prewarmed PBS, and flash-frozen in liquid nitrogen.

#### RNA preparation and reference pool.

P. falciparum RNA sample isolation, cDNA synthesis, labeling, and DNA microarray hybridizations were performed as described by Bozdech et al. (2003). Samples for individual timepoints (coupled to Cy5) were hybridized against a reference pool (coupled to Cy3). The reference pool was comprised of RNA samples representing all developmental stages of the parasite. From this pool, sufficient cDNA synthesis reactions, using 12 μg of pooled reference RNA, were performed for all hybridizations. After completing cDNA synthesis, all reference pool cDNAs were combined into one large pool and then split into individual aliquots for subsequent labeling and hybridization. Microarray hybridizations were incubated for 14–18 h.

#### DNA microarray hybridizations and quality control.

In total, 55 DNA microarray hybridizations covering 46 timepoints were performed. Timepoints 1, 7, 11, 14, 18, 20, 27, and 31 were represented by more than one array hybridization. Data were acquired and analyzed by GenePix Pro 3 (Axon Instruments, Union City, California, United States). Array data were stored and normalized using the NOMAD microarray database system (http://ucsf-nomad.sourceforge.net/). In brief, a scalar normalization factor was calculated for each array using unflagged features with median intensities greater than zero for each channel and a pixel regression correlation coefficient greater than or equal to 0.75. Quality spots were retained based on the following criteria. The log_2_(Cy5/Cy3) ratio for array features that were unflagged and had a sum of median intensities greater than the local background plus two times the standard deviation of the background were extracted from the database for further analysis. Subsequently, expression profiles consisting of 43 of 46 timepoints (approximately 95%) were selected. For those timepoints that were represented by multiple arrays, the ratio values were averaged.

#### FFT analysis of the expression profiles.

Fourier analysis was performed on each profile in the quality-controlled set (5,081 oligonucleotides). Profiles were smoothed with missing values imputed using a locally weighted regression algorithm with local weighting restricted to 12% using R (http://www.R-project.org). Fourier analysis was performed on each profile using the *fft()* function of R, padded with zeros to 64 measurements. The power spectrum was calculated using the *spectrum()* function of R. The power at each frequency (*Power()*), the total power (P_tot_), and the frequency of maximum power (F_max_) were determined. The periodicity score was defined as *Power*[(F_max−1_) + (F_max_) + (F_max+1_)]/P_tot_. The most frequent value of F_max_ across all profiles was deemed the major frequency (m) and used in determining phase information. The phase of each profile was calculated as atan2\[−(I (m)],R (m)\, where atan2 is R's arctangent function and I and R are the imaginary and real parts of the FFT. Profiles were then ordered in increasing phase from −π to π. The loess smooth profiles were drawn through the raw expression data using the *loess()* function found in the modern regression library of R (version 1.5.1). The default parameters were used, with the exception that local weighting was reduced to 30%. For the averaged profiles of the functional groups (see Figure 2B–2M), the loess smooth profiles were calculated for each expression profile individually and subsequently averaged to create the representative profile. These same methods were applied to both the randomized set (see the inset to Figure 1F) and the yeast cell cycle dataset (see Figure S1).

The raw results files (Dataset S1), the fully assembled raw dataset (Dataset S2, the overview dataset (Dataset S3, and the quality control dataset (Dataset S4) are available as downloads.

#### Evaluation of coexpression along chromosomes.

The evaluation of coexpression of genes along chromosomes was carried out as follows. The Pearson correlation coefficient was calculated for each pair of profiles. For ORFs with multiple oligonucleotides, the average profile was calculated. The neighborhood of each ORF profile was defined as a window of between one and ten adjacent ORF profiles. If any window in an ORF profile's neighborhood displayed more than 70% pairwise correlation of greater than 0.75, it was flagged as enriched. The length of the window was then recorded as a region of coexpression. This process was repeated without strand separation of ORFs and with randomly permuted datasets.

#### Comparative genomic hybridization.

P. falciparum strains 3D7 and HB3 were cultured as previously described at a concentration of 10% parasitaemia. Genomic DNA (gDNA) was isolated from a minimum of 500 ml of total culture for each P. falciparum strain, as previously described (Wang et al. 2002). Isolated gDNA from each strain was sheared by sonication to an average fragment size of approximately 1–1.5 kb and then was purified and concentrated using a DNA Clean and Concentrator kit (Zymo Research, Orange, California, United States). Amino-allyl-dUTP first was incorporated into the gDNA fragments with a Klenow reaction at 37°C for 6–8 h with random nonamer primers and 3 μg of sheared gDNA. After purification and concentration of the DNA from the Klenow reaction, CyScribe Cy3 and Cy5 dyes (Amersham Biosciences, Buckinghamshire, United Kingdom) were coupled to HB3 DNA and 3D7 DNA, respectively, as previously described (Pollack et al. 1999). Uncoupled fluorescent dye was removed using a DNA Clean and Concentrator kit. Labeled DNA fragments were hybridized to the oligonucleotide-based DNA microarrays. Fluorescence was detected and analyzed using an Axon Instruments scanner and GenePix Pro 3.0 software. Only features that had median intensities greater than the local background plus two times the standard deviation of the background in each channel were considered for further analysis. For each feature, the percent of the total intensity was determined using the signal in the 3D7 channel as the total amount of intensity for each oligonucleotide; intensity differences less than 50% were considered to be significant for subsequence analysis.

#### Calculation for in-phase plastid-targeted genes.

The range of FFT-based phases for the expression profiles of the plastid genome is between 0.32 and 1.05 (or roughly π/9 −π/3). Using the list of 551 apicoplast-targeted genes available at PlasmoDB.org, we first ordered these genes by phase and then grouped all genes with a phase range between 0.00 and 1.40 (0–4π/9), resulting in 124 genes represented by 128 oligonucleotides on the microarray. This select group represents the in-phase plastid targeted genes (see Table S6).

#### Calculation for vaccine targets.

To select the expression profiles most related to the AMA1, MSP1, MSP3, MSP5, EBA175, RAP1, and RESA1 vaccine candidates, we calculated the similarity of all expression profiles in the dataset to those of these antigens by Euclidian distance. The minimum Euclidian distance calculated for every profile was then binned into 60 bins and plotted as a histogram. A natural break in the histogram was seen that included the set of 262 ORFs (see Figure S2).

## Supporting Information

Dataset S1 Raw GenePix Results (29.5 MB ZIP).

Dataset S2 Complete Dataset (3.7 MB TXT).

Dataset S3 Overview Dataset (2.4 MB TXT).

Dataset S4 Quality Control Set (3.1 MB TXT).

Figure S1 Histogram of the Percent Power at Peak Frequencies for the Yeast Cell Cycle Data The percent of power in the maximum frequency of the FFT power spectrum was used to determine periodicity of the yeast cell cycle data from Spellman et al. (1998). The histogram reveals periodic regulation of gene expression for only a small subset of genes (% power >70%).
(223 KB EPS).

Figure S2 Pearson Correlation Maps for the P. falciparum Chromosomes A matrix of the pairwise Pearson correlations was calculated for every expression profile along the chromosomes. The analysis included all annotated ORFs. The gray areas correspond to a Pearson correlation d(x, y) = 0 and indicate ORFs with no detectable IDC expression or ORFs not represented on the microarray. The starting point (left) and the end point (right) of the chromosomes and the ORF order along the chromosomes are identical to the order in PlasmoDB.org.
(30.9 MB EPS).

Figure S3 CGH of 3D7 versus HB3 for All Chromosomes Genomic differences between strain 3D7 and strain HB3 were measured by CGH. The relative hybridization between the gDNA derived from these two strains is shown as a percent reduction of the signal intensity for 3D7 along individual chromosomes. (1.7 MB ZIP).

(A) (216 KB EPS)

(B) (232 KB EPS)

(C) (237 KB EPS)

(D) (240 KB EPS)

(E) (252 KB EPS)

(F) (232 KB EPS)

(G) (235 KB EPS)

(H) (235 KB EPS)

(I) (249 KB EPS)

(J) (265 KB EPS)

(K) (283 KB EPS)

(L) (270 KB EPS)

(M) (305 KB EPS)

(N) (332 KB EPS)

Figure S4 Distribution of Euclidian Distances between Expression Profiles of the IDC Genes and Seven Vaccine Candidates The similarity between each IDC expression profile and the profiles of the seven selected vaccine candidate genes was evaluated by Euclidian distance calculations, d(x,y) = Σ(x_i_ − y_i_)^2^. The Euclidian distance value to the closest vaccine homologue was selected for each IDC profile and used to generate this plot. Genes with d(x,y) < 20 were selected for the phaseogram of putative vaccine targets (see Figure 5).
(494.02 KB EPS).

Table S1 Pearson Correlation for ORFs Represented by Multiple Oligonucleotides This table contains all of the ORFs in the analyzed dataset that are represented by multiple oligonucleotides on the DNA microarray. The average Pearson correlation value has been calculated for the expression profiles of all oligonucleotides for each given ORF.
(44 KB TXT).

Table S2 P. falciparum Functional Gene Groups This table contains all of the P. falciparum groups discussed. The groups include the following: transcription machinery, cytoplasmic translation machinery, the glycolytic pathway, ribonucleotide synthesis, deoxyribonucleotide synthesis, DNA replication machinery, the TCA cycle, the proteaseome, the plastid genome, merozoite invasion, actin–myosin motility, early-ring transcripts, mitochondrial genes, and the organellar translational group.
(291 KB TXT).

Table S3 Coregulation along the Chromosomes of P. falciparum
This table contains the regions of coregulation found in the chromosomes of P. falciparum determined by calculating the Pearson correlation between expression profiles for contiguous ORFs. The cutoff was 70% pairwise correlation of greater than 0.75 for each group. Only groups of two ORFs or more are listed.
(6 KB TXT).

Table S4 3D7 versus HB3 CGH Data This table contains all of the intensity data from CGH of gDNA derived from the 3D7 and HB3 strains of P. falciparum. The averaged intensities from three microarray hybridization experiments are listed.
(414 KB TXT).

Table S5 Putative Apicoplast-Targeted Genes and Expression Profiles This table contains all of the predicted apicoplast-targeted ORFs from PlasmoDB.org. The presence of each ORF on the DNA microarray is tabulated, as well as whether each ORF is present in the overview set. Finally, the plastid ORFs in-phase with plastid genome expression are listed, as well as the corresponding oligonucleotide identifiers.
(147 KB TXT).

Table S6 Putative P. falciparum Proteases and Their Expression Data The table was constructed by searching the database for any putative protease annotations and contains all of the 92 proteases identified by Wu et al. (2003).
(59 KB TXT).

Table S7 Vaccine Candidate Correlation Table The similarity of all expression profiles to seven known vaccine candidates was evaluated by a Euclidian distance calculation to all expression profiles measured. These 262 ORFs constitute the top 5% of genes in the IDC with minimum distance to these seven ORFs. The seven candidates used are AMA1, MSP1, MSP3, MSP5, EBA175, RAP1, and RESA1.
(204 KB TXT).

## Abbreviations

ASL: adenylosuccinate lyase
CGH: comparative genomic hybridization
CLAG: cytoadherence-linked asexual gene
Clp: caseineolytic protease
DHFR-TS: dihydrofolate reductase–thymidylate synthetase
EBA: erythrocyte-binding antigen
EBL: erythrocyte-binding-like protein
FFT: fast Fourier transform
FP: falcipain
FV: food vacuole
gDNA: genomic DNA
HAP: histo-aspartyl protease
hpi: hours postinvasion
IDC: intraerythrocytic developmental cycle
MSP: merozoite surface protein
ORF: open reading frame
PM: plasmepsin
PV: parasitophorous vacuole
RBC: red blood cell
RBP: reticulocyte-binding protein
RESA: ring-infected surface antigen
SERA: serine repeat antigen
TCA: tricarboxylic acid.
